# Fabrication of an (α-Mn_2_O_3_:Co)-decorated CNT highly sensitive screen printed electrode for the optimization and electrochemical determination of cyclobenzaprine hydrochloride using response surface methodology†

**DOI:** 10.1039/d0ra05106c

**Published:** 2020-07-01

**Authors:** Ahmed M. Abdel-Raoof, Ayman O. E. Osman, Ebrahim A. El-Desouky, Ashraf Abdel-Fattah, Rady F. Abdul-Kareem, Elsayed Elgazzar

**Affiliations:** Pharmaceutical Analytical Chemistry Department, Faculty of Pharmacy (Boys), Al-Azhar University 11751, Nasr City Cairo Egypt Ahmedmeetyazeed79@Azhar.edu.eg Ahmedmeetyazeed79@yahoo.com; Department of Physics, Faculty of Science, Suez Canal University Ismailia Egypt

## Abstract

A new chemically optimized screen-printed electrode modified with a cobalt-doped α-Mn_2_O_3_ nanostructure on carbon nanotube paste (α-Mn_2_O_3_:Co@CNTs) has been constructed for the recognition of cyclobenzaprine hydrochloride. The prepared paste is based on the incorporation of oxide ion conductors, such as the α-Mn_2_O_3_ nanostructure with cobalt and ion pairs (tetraphenyl borate coupled with the drug), as electroactive species in the screen-printed electrode to increase the sensor surface area and decrease electrical resistance. The central composite design is a useful methodology for the estimation and modeling of the exact optimum parameters specifically designed for this process. This is a good way to graphically clarify the relationship between various experimental variables and the slope response. The proposed sensor, α-Mn_2_O_3_:Co@CNTs, possesses very good sensitivity and the ability to recognize the drug over the concentration range of 1 × 10^−6^ to 1 × 10^−2^ mol L^−1^ at 25 ± °C with a detection limit of 2.84 × 10^−7^ mol L^−1^. It exhibits a reproducible potential and stable linear response for six months at a Nernstian slope of 58.96 ± 0.76 mV per decade. The proposed electrode approach has been successfully applied in the direct determination of the drug in its pure and dosage forms.

## Abstract 2

A new chemically optimized screen-printed electrode modified with a cobalt-doped α-Mn_2_O_3_ nanostructure on carbon nanotube paste (α-Mn_2_O_3_:Co@CNTs) has been constructed for the recognition of cyclobenzaprine hydrochloride.

## Graphical abstract

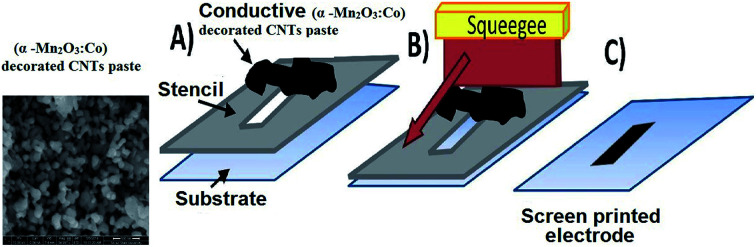

## 1. Introduction

Cyclobenzaprine hydrochloride (CBZ), chemically known as 3-(5*H*-dibenzo[*a*,*d*]cyclohepten-5-ylidene)-*N-N*-dimethyl-1-propanamine hydrochloride,^1^ is a centrally acting muscle relaxant related to tricyclic antidepressants.^2^ CBZ was approved for medical use in the United States in 1977. It is used for muscle spasms from musculoskeletal conditions with sudden onset associated with acute and painful musculoskeletal conditions.^3^ Different analytical methods have been prescribed for the quantitative determination of CBZ, including a non-aqueous titration method in U.S.P.,^4^ spectrophotometric methods,^5–9^ HPLC methods,^10–26^ TLC-densitometric method,^18,27,28^ GC-MS,^29–32^ and the electrochemical method.^33^

Manganese oxide nanoparticles potentially have some remarkably promising applications for sustainable global nanotechnology science.^34^ Owing to their privileged chemical, physical properties and different oxidation states (MnO, Mn_2_O_3_, Mn_3_O_4_, Mn_5_O_8_, and MnO_2_), Mn-oxide NPs can be used in magnetic storage devices, molecular adsorption, solar cells, catalysis, imaging contrast agents, batteries, catalysts, as well as other fields, such as water treatment, medicine, biosensors, optical sensor applications and optoelectronics.^35–39^ Over the last decade, Mn-oxide NPs have found potential applications in ion-exchange, medicine, supercapacitors, electrochemical sensing, and energy storage.^40–42^ MnO-coated sand and MnO_2_-modified clinoptilolite-Ca zeolite have been used for removing arsenic(iii) and arsenic(v) from water.^43,44^ Based on recent research, a graphene-nanosheets/delta-MnO_2_ nanocomposite can be used for disposing of interfering nickel ions from wastewater,^45^ purification of DNA, molecular diagnostics and biochemistry.^46–48^ Graphene–cobalt oxides in a composite film with a graphene electrode are used for the electrochemical sensing of glucose^49,50^ and H_2_O_2_.^51^ Binary cobalt and manganese oxides are employed for electrochemical capacitor applications on activated carbon as advanced oxygen reduction electrocatalysts.^52^ Cobalt oxide-manganese oxide has been employed as chloride ion sensors^53^ and in the amperometric sensing of hydrogen peroxide.^54^ Several technical approaches used in the preparation of dimanganese trioxide (Mn_2_O_3_) include co-precipitation, simple reduction, sol–gel, a microwave process, and hydrothermal methods.^55–57^ In previous work, the catalytic and electrochemical properties of MnO_2_ nanomaterial were explored *via* hydrothermal preparation.^58^ Recently, α-Mn_2_O_3_ nanoparticles were prepared *via* the sol–gel method.^59^ In the current work, manganese sesquioxide (α-Mn_2_O_3_) and cobalt-doped α-Mn_2_O_3_ nanostructures have been prepared by a simple chemical technique known as a co-precipitation method.

Modeling and analysis of the required responses by mathematical and statistical methods are usually applied to identify the optimized responses that are controlled by several independent factors. The experimental design has a great effect on the correctness of RSM to decrease the number of experimental treatments and save time. RSM applications search out the correlation and interactions between responses of interest and the related independent factors.^60^

In this study, experimental design using central composite design (CCD) can explore the correlation between the optimized slope and the most important experimental parameters such as the influence of the nanocomposite content on response optimization. RSM has an essential role in the determination of the optimum independent parameters with low cost, high sensitivity and short time without any derivatization, or extraction procedure.

## 2. Experimental

### 2.1. Apparatus

pH-meter Jenway 3510 (England), Benchtop centrifuge (TDL-60B) (Hunan, China, Mainland), Bandelin sonorex, Rx 510 S, magnetic stirrer (Hungarian). Hot plate (Torrey pines Scientific, USA). Dimanganese trioxide (α-Mn_2_O_3_) and cobalt-doped dimanganese trioxide (α-Mn_2_O_3_:Co) samples were examined by scanning electron microscopy (SEM; Helios Nanolab 400) with energy dispersive X-ray analysis (EDX; Model, Helios Nanolab 400) attachment, Raman spectra (Horiba Lab RAM HR Evolution) and X-ray diffraction (XRD) (Model, Rigaku Smart Lab.). The prepared samples were calcined in a furnace (DELTA-MF06, India). The optimization and design of the proposed method were performed by the Design-Expert® trial version 11.0 software.

### 2.2. Materials and chemicals

CBZ powder was kindly provided by Global Napi Pharmaceutical Company, 6-October City, Egypt. Its purity was 99.55 ± 0.15% (Batch no. 0247564). Moveasy® 10 mg tablets, claimed to contain 10 mg CBZ, manufactured by Global Napi Pharmaceuticals Company, 6-October City, Batch o. F27902 and purchased from the local pharmacy. Acetone, nitric acid (55.5% w/w), citric acid, glycine, glucose, urea, glycine, sucrose sodium hydroxide, boric and sulfuric acid (98% w/w) were obtained (El-Nasr Company, Egypt). Polyvinylchloride (PVC), sodium tetraphenyl borate (TPB), phosphoric, acetic acids, high purity graphite powder (10–20 μm), cyclohexanone and multi-wall carbon nanotube powder (carbon > 95.0%, O.D. × L 6–9 nm *×* 5 μm) were obtained from (Sigma-Aldrich, Germany). Calcium chloride, sodium chloride, nickel chloride hexahydrate, magnesium chloride, manganese chloride and cobalt chloride (Prolabo, Paris, France).

### 2.3. Standard drug solutions

A stock standard solution of CBZ (1 × 10^−2^ mol L^−1^) was prepared in a 100 mL calibrated glass flask by dissolving 311.9 mg of CBZ powder in 60 mL of double distilled water and made up to the volume with the same solvent. Working solutions (1 × 10^−3^–1 × 10^−7^ mol L^−1^) were freshly prepared by two methods, either in Britton Robinson buffer (B–R)^61^ for the pH effect study or, in double-distilled water for the calibration and other studies after serial dilution from the stock solution.

### 2.4. Procedures

#### 2.4.1. Preparation of dimanganese trioxide (α-Mn_2_O_3_) and cobalt-doped dimanganese trioxide (α-Mn_2_O_3_:Co) by the co-precipitation method

Manganese chloride (MnCl_2_), cobalt chloride (CoCl_2_) and sodium hydroxide (NaOH) have been used as starting materials in the preparing process. For synthesizing dimanganese trioxide (α-Mn_2_O_3_), a desired amount of MnCl_2_ was dissolved in 20 mL of distilled water for 2 hours using a magnetic stirrer. Then, 20 mL NaOH solution was carefully added dropwise to the aqueous solution under vigorous stirring for another 3 hours without heating, to ensure that a homogeneous solution was formed. The product precipitate was filtered and washed many times with deionized water to remove all residual ions, then dried overnight in a furnace at 80 °C and finally annealed at 450 °C. Cobalt-doped dimanganese trioxide (Mn_2_O_3_:Co) was prepared by dissolving 0.5 g cobalt chloride in 10 mL deionized water and subsequently adding it drop by drop to manganese chloride solution with stirring for 3 hours. Next, 20 mL NaOH solution was carefully added dropwise to the mixture of manganese–cobalt chloride to make sure that the precipitant was composed. The final product was filtered and washed many times with deionized water to remove all residual ions, then dried in a furnace at 80 °C for 24 h and finally annealed at 450 °C.

#### 2.4.2. Preparation of carboxylated multi-walled carbon nanotubes

The reaction mixture containing 1.5 g MWCNTs was refluxed with dilute H_2_SO_4_ + HNO_3_ (3 : 1) at 55 °C for 12 hours and stirred at 40 °C for another 12 hours; the resulting suspension was centrifuged and filtered. The resulting carboxylated MWCNTs (MWCNT-COOH) were washed several times and dried under vacuum at 60 °C for 16 hours.^62^

#### 2.4.3. Preparation of the ion associate

The ion associate (CBZ–TPB) was prepared by a dropwise addition of equal volumes (30 mL) and concentrations (1 × 10^−2^ mol L^−1^) of both CBZ and TPB solutions. The obtained precipitate was allowed to coagulate for two days, then filtered and washed several times with distilled water. Finally, the remaining precipitate was dried under vacuum oven at 60 °C for 4 hours.

### 2.5. RSM optimization

The central composite design (CCD) has been widely employed for process optimization and is generally used for fitting quadratic surface models well suited to process optimization. The functionalized carbon nanotube amount, an ion pair content and Mn_2_O_3_:Co NPs amount, as critical independent method parameters, are presented in (Table 1S†). A central composite design (CCD) with 20 experimental conditions (Table 2S†) was generated. The fitted model was obtained through the transformation option as shown in (Table 3S†). Further optimization was performed to predict the ideal paste amount for an optimized response.

### 2.6. Sensor fabrication

The screen printing process can be constructed on an X-ray sheet pre-stressed on a wooden frame as mentioned before in the previous work.^62^ The bare electrode (sensor 1) was fabricated by thoroughly incorporating 50 mg of CNTs and 68 mg of CBZ–TPB ionophores with 1 g graphite powder and mixing well in 0.50 g paraffin oil, then adding 2 g of PVC solution drop-wise to obtain a uniform paste. A functionalized paste (second sensor) was prepared similarly to sensor 1 and modified with the addition of an appropriate amount of (MWCNT-COOH) modified with Mn_2_O_3_ NPs. Sensor 3 was prepared as before plus the addition of 50 mg Mn_2_O_3_:Co to prepare modified α-Mn_2_O_3_:Co@CNTs as shown as (Table 1). The proposed sensors were printed and dried under vacuum at 50 °C for 60 minutes and subjected to preconditioning in their stock solutions before starting direct calibrations.

**Table 1:** Different compositions of the optimal paste for different proposed electrodes

Electrode type	Composition					
	Ion pair amount (mg)	Graphite (mg)	Functionalized CNTs (mg)	Mn_2_O_3_-NPs (mg)	α-Mn_2_O_3_:Co-NPs (mg)	Plasticizer (mg)
Electrode 1	68	1000	50	—	—	550
Electrode 2	68	950	50	50	—	550
Electrode 3	68	950	50	—	50	550

### 2.7. Sensor calibration

The working sensors were immersed in different solutions covering the concentration range of 1 × 10^−7^ to 1 × 10^−2^ mol L^−1^ CBZ. The potential readings were recorded and plotted *versus* CBZ ion activity in the logarithmic scale for electrode performance evaluation according to IUPAC recommendations.^63^

### 2.8. Pharmaceutical sample analysis

Recovery studies were carried out by the standard addition technique to confirm the specificity of the suggested method by adding 20 mL aliquots (10^−5^ to 10^−3^ mol L^−1^) of CBZ solution to the same volume of pharmaceutical solution (10^−3^ mol L^−1^) and the change in mV reading after each incremental addition was recorded to calculate the drug concentration using the following equation:1
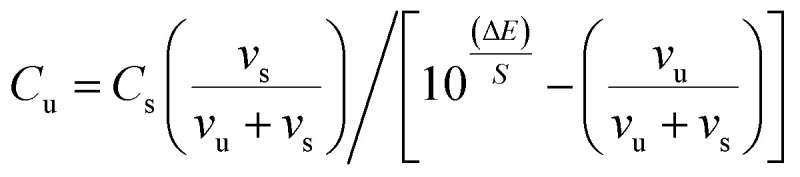


## 3. Results and discussion

### 3.1. Characterization

**Fig. 1:**
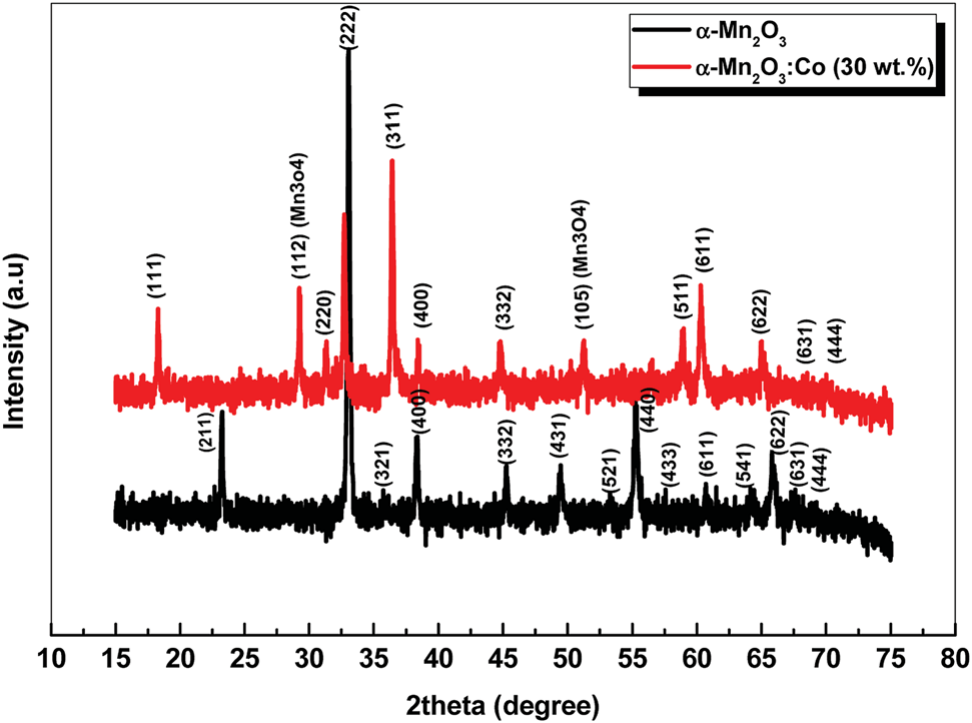
XRD of α-Mn_2_O_3_ and α-Mn_2_O_3_:Co (30 wt%) nanoparticles calcined at 450 °C.

XRD was carried out to investigate the phase structure and crystallinity of the synthesized α-Mn_2_O_3_ and Co-doped α-Mn_2_O_3_ nanostructure calcined at 450 °C. Fig. 1 displays diffraction peaks at 2*θ* positions 23.167°, 33.022°, 35.755°, 38.397°, 45.308°, 49.566°, 53.43°, 55.361°, 57.49°, 60.735°, 64.402°, 66.02°, 67.647° and 68.96°, corresponding to (*hkl*) planes (211), (222), (321), (400), (332), (431), (521), (440), (433), (611), (541), (622), (631) and (444), respectively, in good agreement with the standard α-Mn_2_O_3_/cubic phase (JCPDS card no. 71-0636).^64^ No other characteristic peaks of impurities or the Mn_3_O_4_ phase were detected in the XRD pattern, demonstrating the pure phase of the product, and the strong characteristic diffraction peak at 2*θ* = 33.02° confirmed that the α-Mn_2_O_3_ cubic structure was formed. On the other hand, (α-Mn_2_O_3_:Co 30 wt%) shows weak peaks of low crystallinity shifted to lower angles, which indicate the influence of the Co doping concentration on the α-Mn_2_O_3_ structure. The diffraction peaks at 18.29° (111), 31.28° (220), 36.47° (311) and 59.01° (511) are attributed to tricobalt tetraoxide (Co_3_O_4_) (JCPDS no. 42-1467) owing to the difference in the electronegativity of Co (1.90) and Mn (1.50) and also the high concentration of cobalt (30 wt%) inside the Mn_2_O_3_ lattice. It can be seen that another phase of Mn_3_O_4_ has been depicted in the spectrum at 2*θ* = 29.16° and 51.20° because of the high electrochemical activity of Mn_3_O_4_. The average crystallite size (*D*) of the as-synthesized samples was estimated by applying the Debye–Scherrer equation from the most intense peak (222).^65^2
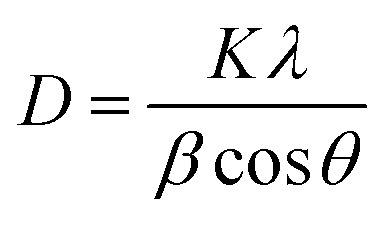
*D* is the average nanocrystalline diameter, *β* is the full width at half maximum FWHM, *λ* is the wavelength of incident X-ray, *K* = 0.94 is the shape factor and *θ* denotes the diffraction angle. The crystallite size of α-Mn_2_O_3_ and Co-doped α-Mn_2_O_3_ was calculated at 40 nm and 47 nm, respectively. The dislocation density (*δ*) defined as the length of the dislocation line per unit volume of the crystal can be estimated by Williamson and Smallman's relation:^66^3
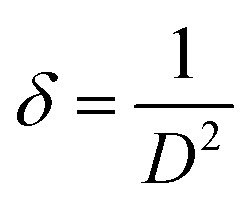
where *D* is the grain size of the crystal. The dislocation densities of 6.25 × 10^−4^ nm^−2^ and 4.5 × 10^−4^ nm^−2^ were obtained for α-Mn_2_O_3_ and Co-doped α-Mn_2_O_3_, respectively, indicating the existence of few lattice defects and good crystallinity.

**Fig. 2:**
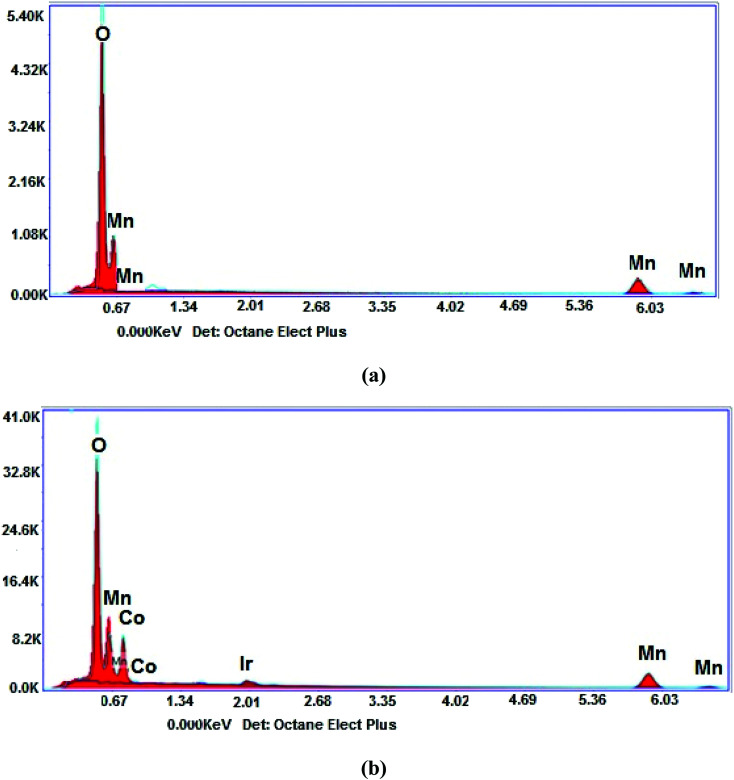
EDX spectra of (a) α-Mn_2_O_3_ and (b) α-Mn_2_O_3_:Co (30 wt%) nanoparticles calcined at 450 °C.

The elemental composition of the nanostructure is demonstrated in Fig. 2(a and b). As can be seen in Fig. 2(a), the spectrum of α-Mn_2_O_3_ depicts Mn and O elements with weight% of 67.95 and 32.05, respectively. Fig. 2(b) shows the Co element appearing at 0.776 keV with weight% of 12.65, which clearly confirmed the incorporation of Co atoms inside the α-Mn_2_O_3_ lattice. The Ir element observed in the spectrum is due to the coating of samples through the measuring process.

**Fig. 3:**
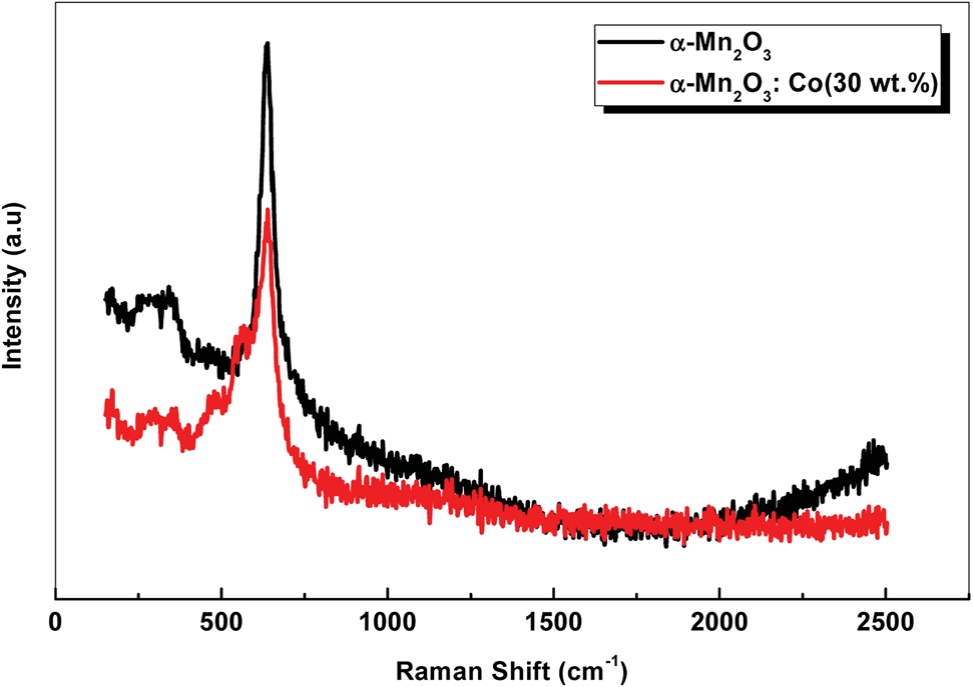
Raman spectra of α-Mn_2_O_3_ and α-Mn_2_O_3_:Co (30 wt%) nanostructure.

Raman scattering spectra of the samples are shown in Fig. 3. α-Mn_2_O_3_ exhibits two peaks at 337 cm^−1^ and 636 cm^−1^. The weak peak at 337 cm^−1^ is assigned to Mn–O vibrations and the other peak at 636 cm^−1^ is attributed to Mn–O–Mn stretching. On the other hand, Co-doped α-Mn_2_O_3_ shows five peaks at 294 cm^−1^, 364 cm^−1^, 485 cm^−1^, 566 cm^−1^ and 640 cm^−1^. A shift of about 4 cm^−1^ was observed for the sharp peak at 640 cm^−1^ owing to the change in the Fermi level inside the optical band gap due to the doping process.

**Fig. 4:**
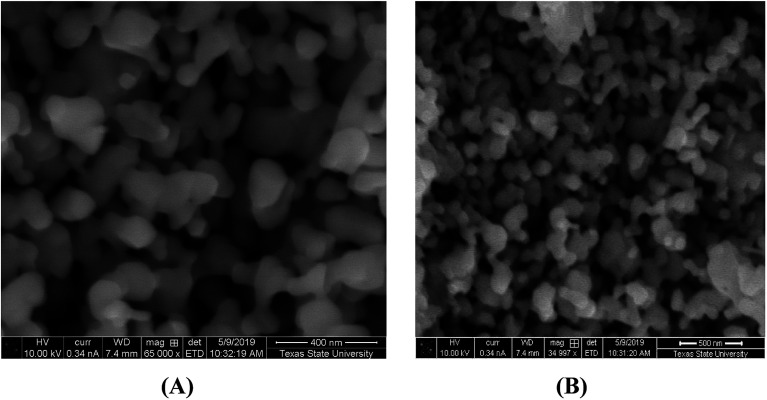
SEM images of (A) α-Mn_2_O_3_ and (B) α-Mn_2_O_3_:Co (30 wt%) nanopowder annealed at 450 °C.

SEM images of the synthesized nanoparticles are depicted in Fig. 4(A and B). It is clear from Fig. 4(A) that α-Mn_2_O_3_ is uniformly spread in a spherical shape with an average particle size of 60 nm. Fig. 4(B) presents Co-doped α-Mn_2_O_3_ with high-density needle particles in the nanoscale range. In addition, the surface morphology appears to be rough due to the large agglomerations of the particles.

### 3.2. Sensor performance assessment

**Fig. 5:**
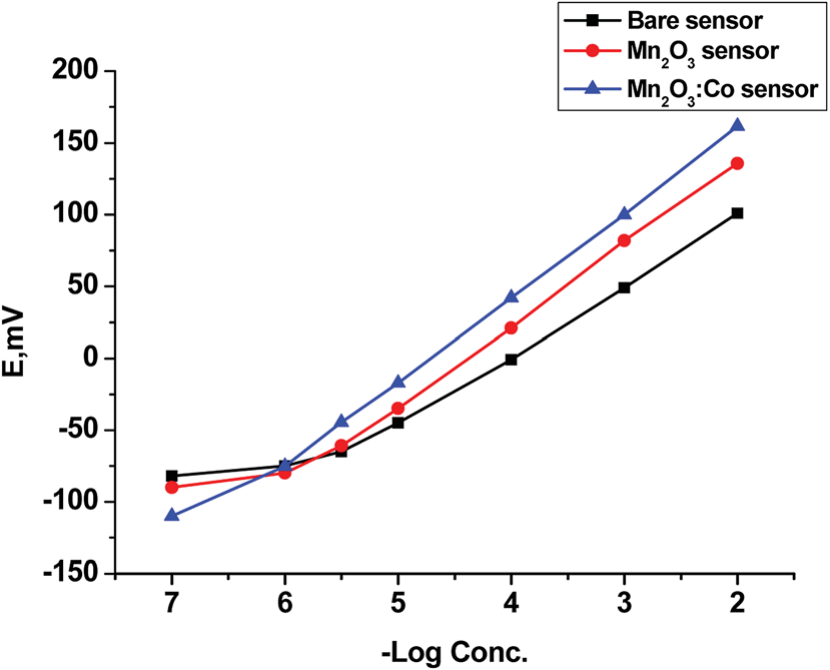
Profile of the potential in mV *versus* −log molar concentration of CBZ for different SPEs.

The proposed sensors can be effectively used for the potentiometric determination of CBZ owing to the results obtained from a potentiometric response of all developed electrodes over the concentration ranges of 1 × 10^−5^–1 × 10^−2^, 5 × 10^−6^–1 × 10^−2^, 1 × 10^−6^–1 × 10^−2^ mol L^−1^ for sensors 1, 2, and 3, respectively. The proposed sensors were evaluated according to IUPAC recommendations.^63^ Critical response characteristics of the bare sensor were improved by the addition of α-Mn_2_O_3_ to the paste where α-Mn_2_O_3_:Co-SPE was the best with a lower detection limit of 2.84 × 10^−7^ mol L^−1^, wide linear range and good correlation coefficient as compared to the other two electrodes (Table 2). Further study was conducted on α-Mn_2_O_3_:Co-SPE related to its good performance and response (Fig. 5).

**Table 2:** Critical response characteristics of different proposed sensors

Parameter	Bare SPE	α-Mn_2_O_3_@CNTs SPE	α-Mn_2_O_3_:Co@CNTs SPE
Slope (mV per decade)	−48.80	−56.85	−58.96
Intercept (mV)	196.80	250.33	278.45
Concentration range (mol L^−1^)	1 × 10^−5^–10^−2^	5 × 10^−6^–10^−2^	1 × 10^−6^–1 × 10^−2^
LOD (mol L^−1^)	7.21 × 10^−6^	1.77 × 10^−6^	2.84 × 10^−7^
Correlation coefficient	0.998	0.9995	0.9997
Response time (s)	15	10	10
Working pH range	4–8	4–8	4–8
Stability (months)	4	4	5
Accuracy (% *R*)	100.45	98.14	99.77
Precision (% RSD)			
Repeatability	1.464	0.887	0.784
Intermediate precision	1.654	0.776	0.651
Robustness (mean ± % RSD)	100.92 ± 1.066	98.66 ± 1.666	99.32 ± 0.675

### 3.3. Response surface and tolerance analysis

The quadratic model is the best-fitted one (Table 3S†). The addition of the quadratic (squared) terms to the model is significant, based on the data analysis related to CCD (Table 4S†). Consequently, a quadratic equation used to fit the data to find the relationship between the critical method parameters and related method attributes by building the predictive model.

The mathematical optimization of the prediction model follows the next equation, representing the linear, quadratic, and interaction terms related to its response.

4 Slope = 58.29 + 0.458*x*_1_ + 0.252*x*_2_ + 0.820*x*_3_ + 0.149*x*_1_^2^ − 0.698*x*_2_^2^ − 0.239*x*_3_^2^ + 0.463*x*_1_*x*_2_ − 0.887*x*_1_*x*_3_ + 0.088*x*_2_*x*_3_

where *x*_1_, *x*_2_, and *x*_3_ are α-Mn_2_O_3_:Co-NPs, functionalized CNTs and ion pair amounts, respectively.

ANOVA data analysis for the quadratic model revealed that a lack of fit is insignificant for the model, which fits well in the experimental design as the *p*-value of lack of fit should be non-significant (*p* > 0.05). The predicted *R*^2^ indicated that the statistics identified cases where the model provides a good fit for the existing data, or not, and its value was 0.9679. The adjusted *R*^2^ compares models with different numbers of variables and its value was 0.9885, which is acceptably compatible with the predicted *R*^2^. Adequate precision was 47.14, where it should not be less than 4. It was concluded from the result obtained that the model was well fitted for experimental design (Table 5S†).

### 3.4. The statistically fitted model diagnostics

The normal probability plot of the residuals is a special case of the probability plot for clarifying that the relationship between the theoretical values and the observed values is approximately linear; it recommends that the error terms are undoubtedly normally distributed. Externally calculating the residuals plot was more effective in detecting outliers and in assessing the equal variance assumption, as shown in Fig. 1S,† revealing that there were no obvious outliers or unusual observations, and the residuals were randomly distributed *versus* the run number. Cook's distance explores the observations that greatly affect the fitted values of the model. The accuracy outcomes of the data obtained from the regression model are acceptable because nothing stands out in the chart as shown in Fig. 1S.†

### 3.5. Setting the optimization criteria

For any given response, a useful class of desirability functions was proposed by Harrington and Derringer to simultaneously optimize the multiple responses and improved the practicality.^67^ Multi-response optimization techniques in practice are usually assessed by the desirability function approach. Multiple-response optimization means that more than one optimization criterion is considered at the same time. In order to do so, one needs to convert the results of the different criteria (method parameters optimization and an optimized slope) into one scalar value. The approach works in two steps. Firstly, functions from 0 to 1 will project the output variables to a value between 0 and 1, with 0 being very bad and 1 being ideal. Depending on the type of optimization, the ramp function will try to hit a target value (slope = 59.00 mV per decade). Secondly, multiply the ramp values of the different criteria by each other. The resultant value of the so-called desirability function will be in the range of zero and 1.0.

Numerical optimization, overlay plots and 3D plots exhibit that we can obtain the desirability equal one by using 50 mg functionalized carbon nanotubes, 50 mg Mn_2_O_3_:Co@CNTs and 68 mg of an ion pair (Fig. 2S–4S†). The practical value will be less than one, but the desirability function itself is typically smooth enough to be optimized with fairly simple algorithms (Table 6S†).

### 3.6. Optimization conditions

#### 3.6.1. Soaking time effect

**Fig. 6:**
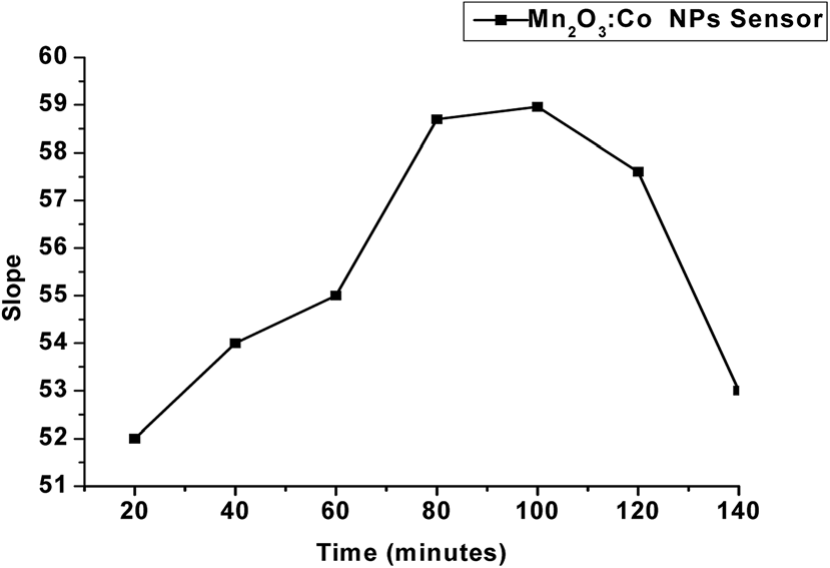
Effect of soaking time on the response of α-Mn_2_O_3_:Co@CNTs SPE.

The effect of soaking time on the performance of freshly prepared α-Mn_2_O_3_:Co@CNTs SPE surfaces was studied to activate the membrane surface. It was performed by measuring the slope of the calibration graphs at different intervals starting from 20 minutes up to 140 minutes. The optimum soaking time was found to be 100 minutes with an optimum slope of −58.96 mV per concentration decade and concentration range of 1 × 10^−6^ to 1 × 10^−2^ mol L^−1^. A longer soaking time above 100 minutes is not recommended as it negatively affects the response of the electrode due to the slow loss of the active membrane (Fig. 6).

#### 3.6.2. Effect of pH

**Fig. 7:**
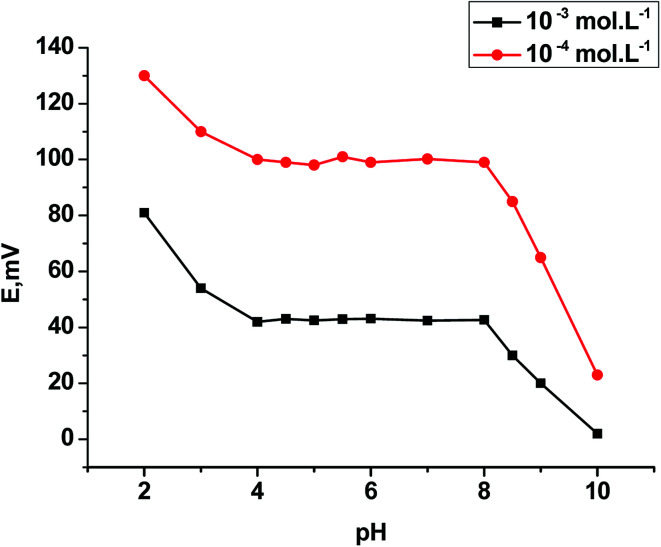
Effect of pH on the response of α-Mn_2_O_3_:Co@CNTs SPE.

The effect of the pH of the test solutions on the potentiometric performance of α-Mn_2_O_3_:Co@CNTs SPE was evaluated by soaking the selected electrode in both concentrations (1 × 10^−4^ and 1 × 10^−3^ mol L^−1^ CBZ) over the pH range 2–10. The selected electrode gave a constant potential over the pH range 4–8. At lower pH, less than 4, the electrode potential reading in mV was increased due to the interference of the hydronium ions in the gel layer of the membrane. For pH greater than 8, the electrode potential reading began to decrease due to the dehydronation of CBZ by unprotonated species leading to a gradual decrease in its concentration (Fig. 7).

#### 3.6.3. Determination of selectivity coefficient

The selectivity coefficient values were evaluated by α-Mn_2_O_3_:Co-SPE to study the effect of interfering cations, employing either the separate solution method^68^ for inorganic cations, or by a matched potential method for several nitrogenous compounds.^69^

The selectivity coefficient values reflect that inorganic cations have no interference due to the difference in ionic size and subsequently, their permeability and mobility when compared to the CBZ cation. In the case of other organic compounds, the selectivity is related to a difference in polarity, *i.e.*, the variations in their polarity relative to the CBZ ion (Table 3).

**Table 3:** Selectivity coefficients of various interfering species for α-Mn_2_O_3_:Co@ CNTs SPE

aInterfering ion (10^−3^ mol L^−1^)	log *K*	
	bSSM	cMPM
KCl	−2.12	—
NaCl	−2.65	—
MgCl_2_	−3.51	—
CaCl_2_	−2.77	—
Nicl_2_·6H_2_O	−3.34	—
Sucrose	—	−2.23
Urea	—	−2.54
Glucose	—	−3.72
Glycine	—	−3.64
Citric acid		−2.75

a All interfering ions are 1 × 10^−3^ mol L^−1^.

b SSM: separate solution method.

c MPM: matched potential method.

#### 3.6.4. Storage stability

In order to prevent humidity-induced damage, the electrodes should be stored under climatically controlled conditions. The selected sensor was strongly used for at least 15 successive measurements and the recommended maximum storage time for α-Mn_2_O_3_:Co-SPE was six months.

## 4. Method validation

IUPAC recommendations are intended to provide requirements and recommendations for the proposed method validation.

### 4.1. Linearity and limit of detection (LOD)

The regression plot was found to be linear over the concentration range of 1 × 10^−6^ to 10^−2^ mol L^−1^ for α-Mn_2_O_3_:Co-SPE (Table 2).

LOD was calculated according to the IUPAC recommendation from the intersection point of the extrapolated mid-range linear segment and the limiting high activity response. The values are given in Table 2.

### 4.2. Accuracy and precision

The accuracy of the suggested method using α-Mn_2_O_3_:Co-SPE for the estimation of CBZ refers to how close measurements are to the theoretical value and is assessed as percent recovery. The precision refers to how close two or more measurements are to each other, whether those measurements are accurate or not, and is assessed as percent relative standard deviation. The data obtained from the proposed method exhibited high accuracy and precision as summarized in Table 2.

### 4.3. Robustness

Robustness measures the flexibility of the proposed method with the slight deliberate variation in certain method parameters by changing one parameter while the other remains fixed, such as a slight change in pH (±0.1) or the soaking time (±2 minutes). No influence on the results was determined by the analytical method, confirming the robustness of the procedure.

### 4.4. Analytical applications

The response characteristics of the selected optimized sensor were investigated by the standard addition technique for the selective electrochemical determination of CBZ in its tablet form. The results are summarized in Table 4. The results of the statistical comparison of the proposed potentiometric method using the selected optimized sensor and the reported method^33^ for the analysis of CBZ in Moveasy® tablets were evaluated and appeared to be suitable for the routine determination of CBZ in pharmaceutical preparation as illustrated in Table 5.

**Table 4:** Results of the recovery study of CBZ *via* the standard-addition method

Sample	α-Mn_2_O_3_:Co@CNTs SPE		
Pure solution (mol L^−1^)	aRecovery%	Moveasy® tablets (mol L^−1^)	aRecovery%
1 × 10^−5^	98.43	1 × 10^−5^	98.56
5 × 10^−5^	99.08	5 × 10^−5^	98.43
1 × 10^−4^	100.78	1 × 10^−4^	99.65
5 × 10^−4^	98.66	5 × 10^−4^	98.98
1 × 10^−3^	101.2	1 × 10^−3^	101.03
Mean ± % RSD	99.63 ± 1.276		99.33 ± 1.069

a Recovery of 3 determinations.

**Table 5:** Results of the statistical analysis of tablets by the proposed α-Mn_2_O_3_:Co@CNTs SPE and the reported method

Parameter	α-Mn_2_O_3_:Co@CNTs SPE	Reported method^33^
*N*	5	5
Mean	98.55	99.96
SD	0.654	0.788
Variance	0.428	0.621
Student's *t*-test	1.433 (2.306)^a^	
*F*-value	1.451 (6.338)^a^	

a The values in parentheses are the corresponding tabulated *t* and *F* values at *P* = 0.05.

## 5. Conclusion

A green chemistry approach has been effectively used for the electrochemical detection of MRB using the α-Mn_2_O_3_:Co@CNTs fabricated sensor. The present work illustrates the tendency of Mn_2_O_3_ to be doped with cobalt nanostructure on a carbon nanotube paste, owing to its high magnetic electronic properties, biocompatibility, catalytic electrical conductivity, and subsequently increase the sensitivity of the electrode with a very low detection limit as compared to other electrodes. The interference and selectivity studies revealed that the present system has good selectivity for MRB in the presence of interfering moieties. The fabricated potentiometric sensor using α-Mn_2_O_3_:Co@CNTs explores high sensitivity, accuracy, precision, fast static response, reasonable selectivity and long-term stability.

## Conflicts of interest

There are no conflicts to declare.

## Supplementary Material

RA-010-D0RA05106C-s001
