# Supplementary material for: Fabrication of an (α-Mn2O3:Co)-decorated CNT highly sensitive screen printed electrode for the optimization and electrochemical determination of cyclobenzaprine hydrochloride using response surface methodology
Source: RSC Adv. 2020 Jul 1;10(42):24985–93. doi: 10.1039/d0ra05106c (PMC9055185; doi:10.1039/d0ra05106c)
Supplement: RA-010-D0RA05106C-s001 [file RA-010-D0RA05106C-s001.pdf]

Electronic Supplementary Material (ESI) for RSC Advances.
This journal is © The Royal Society of Chemistry 2020

Table 1S: Build information of variables specification and experimental domain factors

Factor	Name	Units	Minimum -α	Maximum +α	Coded Low (-1)	Coded High (+1)	Mean Centre point (0)
X_1	**α-Mn_2O_3:Co@CNTs**	mg	23.18	56.82	30.00	50.00	40.00
X_2	Functionalized CNTs	mg	6.36	73.64	20.00	60.00	40.00
X_3	Ion pair	mg	56.59	73.41	60.00	70.00	65.00

Table 2S: Central composite design (CCD) for experimental variables and slope response

		**Factor 1**	**Factor 2**	**Factor 3**	**Response 1**
**Std**	**Run**	**A α-Mn_2O_3:Co@CNTs**	**B:Functionalized CNTs**	**C:Ion pair**	**Slope**
		**mg**	**mg**	**mg**	**mV/decade**
1	15	30	20	60	55.5
2	9	50	20	60	57.4
3	11	30	60	60	55
4	7	50	60	60	58.6
5	1	30	20	70	58.8
6	6	50	20	70	57
7	17	30	60	70	58.5
8	2	50	60	70	58.7
9	3	23.1821	40	65	58.1
10	8	56.8179	40	65	59.5
11	13	40	6.36414	65	56
12	4	40	73.6359	65	56.8
13	14	40	40	56.591	56.3
14	10	40	40	73.409	59.1
15	19	40	40	65	58.2
16	18	40	40	65	58.2
17	16	40	40	65	58.3
18	20	40	40	65	58.3
19	5	40	40	65	58.5
20	12	40	40	65	58.2

Table 3S: fitting summary for the suggested experimental design.

Source	Sequential p-value	Lack of Fit p-value	Adjusted R^2	Predicted R^2	
Linear	0.0231	< 0.0001	0.3340	0.0073	
2FI	0.0289	< 0.0001	0.5810	0.3569	
**Quadratic**	**< 0.0001**	**0.3086**	**0.9885**	**0.9679**	**Suggested**
Cubic	0.8540	0.0623	0.9843	0.4119	Aliased

Warning: The Cubic model is aliased.

Table 4S ANOVA for Quadratic model

Source	Sum of Squares	Mean Square	F-value	p-value	
**Model**	29.27	3.25	182.85	< 0.0001	**significant**
A-α-**Mn_2O_3:Co**@CNTs	2.86	2.86	161.03	< 0.0001	
B- Functionalized CNTs	0.8692	0.8692	48.87	< 0.0001	
C-Ion pair	9.20	9.20	517.21	< 0.0001	
AB	1.71	1.71	96.20	< 0.0001	
AC	6.30	6.30	354.25	< 0.0001	
BC	0.0612	0.0612	3.44	0.0932	
A^2	0.3228	0.3228	18.15	0.0017	
B^2	7.04	7.04	395.70	< 0.0001	
C^2	0.8249	0.8249	46.37	< 0.0001	
**Residual**	0.1779	0.0178			
Lack of Fit	0.1095	0.0219	1.60	0.3086	**not significant**
Pure Error	0.0683	0.0137			
**Cor Total**	29.45				

The **Model F-value** of 182.85 implies the model is significant. There is only a 0.01% chance that an F-value this large could occur due to noise.

P-values less than 0.0500 indicate model terms are significant. In this case A, B, C, AB, AC, A^2, B^2, C^2 are significant model terms.

Table 5S Fit Statistics

**Std. Dev.**	0.1334	**R^2**	0.9940
**Mean**	57.75	**Adjusted R^2**	0.9885
**C.V. %**	0.2309	**Predicted R^2**	0.9679
		**Adeq Precision**	47.1430

The **Predicted R^2** of 0.9679 is in reasonable agreement with the **Adjusted R^2** of 0.9885; i.e. the difference is less than 0.2.

Adeq Precision measures the signal to noise ratio. A ratio greater than 4 is desirable. Your ratio of 47.143 indicates an adequate signal.

Table 6S: Solutions determination of optimum response.

Number	α-Mn_2O_3:Co@CNTs	Functionalized CNTs	Ion pair	Slope	Desirability	
1	49.389	46.809	64.591	59.000	1.000	
2	35.844	45.878	69.602	59.000	1.000	
3	**49.660**	**50.038**	**67.255**	**59.000**	**1.000**	**Selected**
4	49.782	47.676	62.630	59.000	1.000	
5	30.521	24.294	69.743	59.000	1.000	
6	45.660	47.039	69.252	59.000	1.000	
7	36.057	45.290	69.623	59.000	1.000	
8	49.316	49.127	66.491	59.000	1.000	
9	49.302	50.729	64.379	59.000	1.000	
10	49.620	50.089	67.196	59.000	1.000	

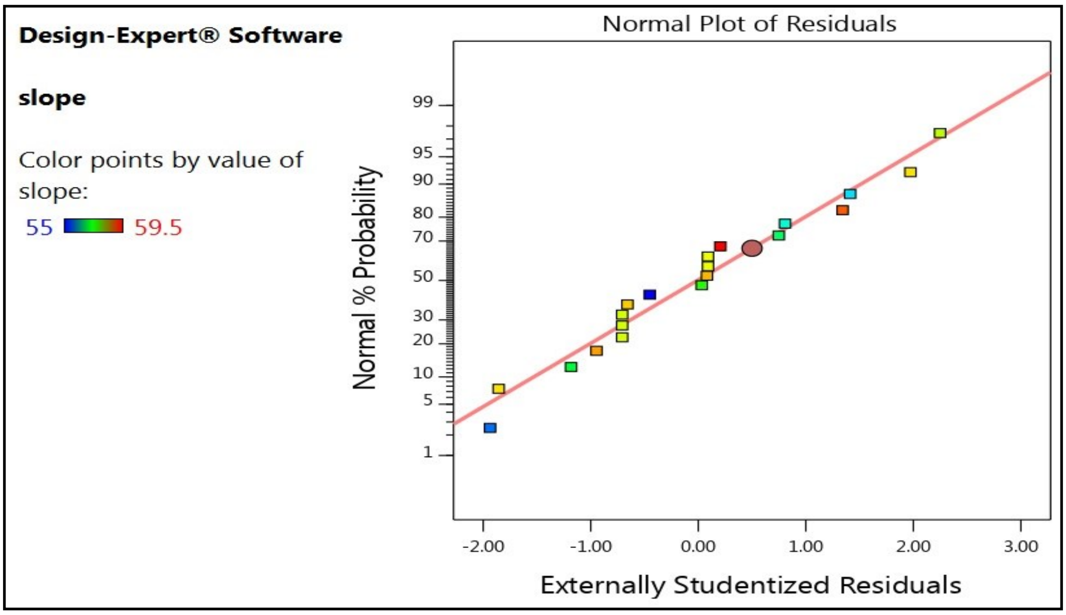

(A)

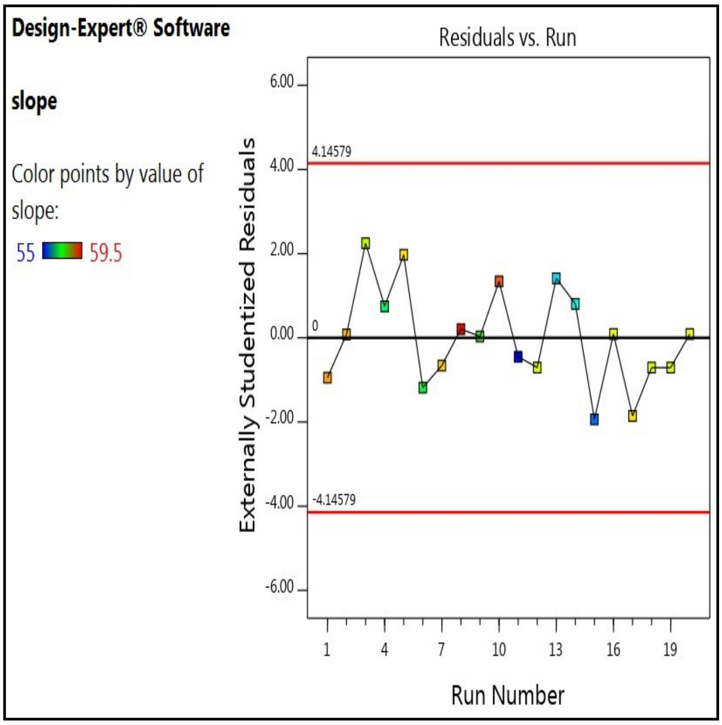

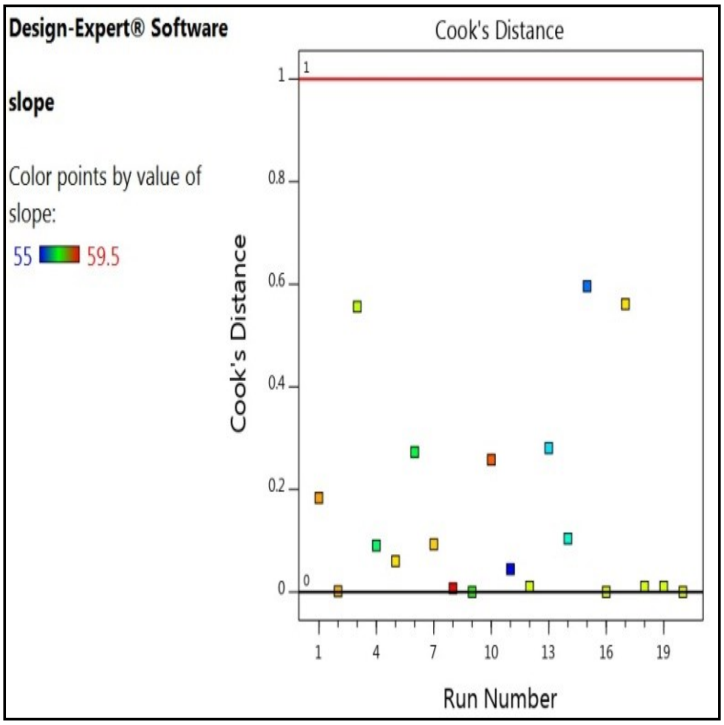

(B) (C)

Fig. 1S: Normal probability plot of the residuals (A), residuals versus run (B), cook's distance (C).

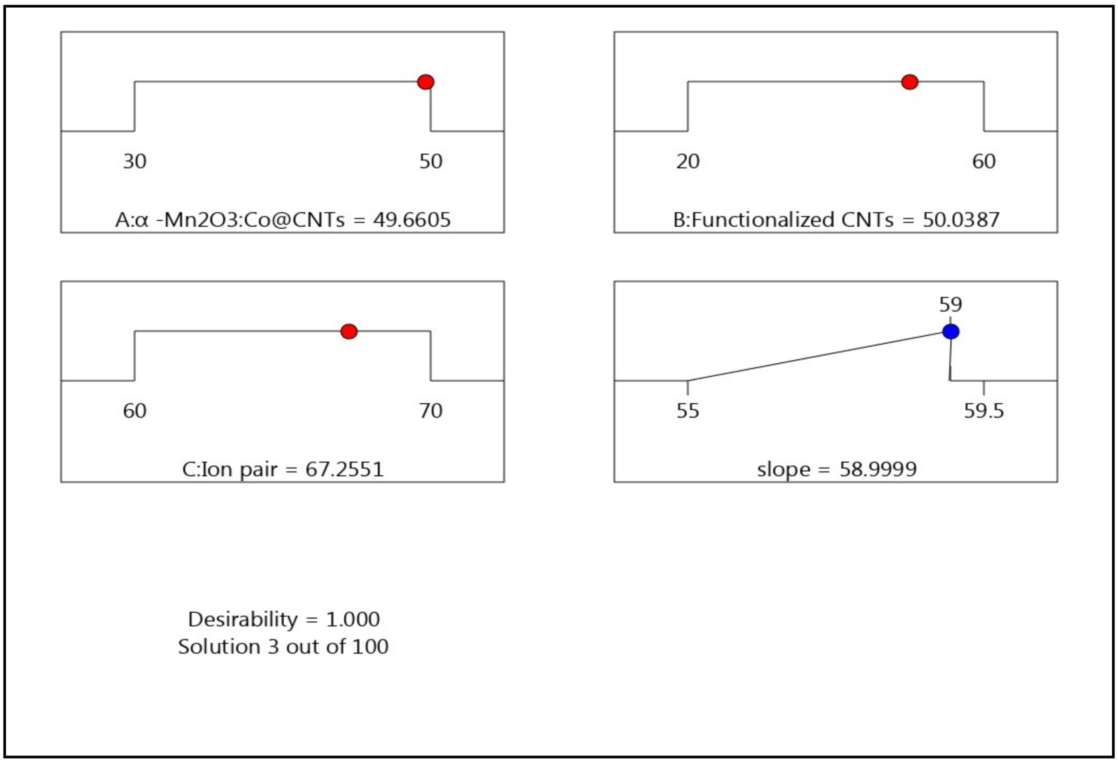

Fig. 2S: Numerical optimization ramps view for solutions.

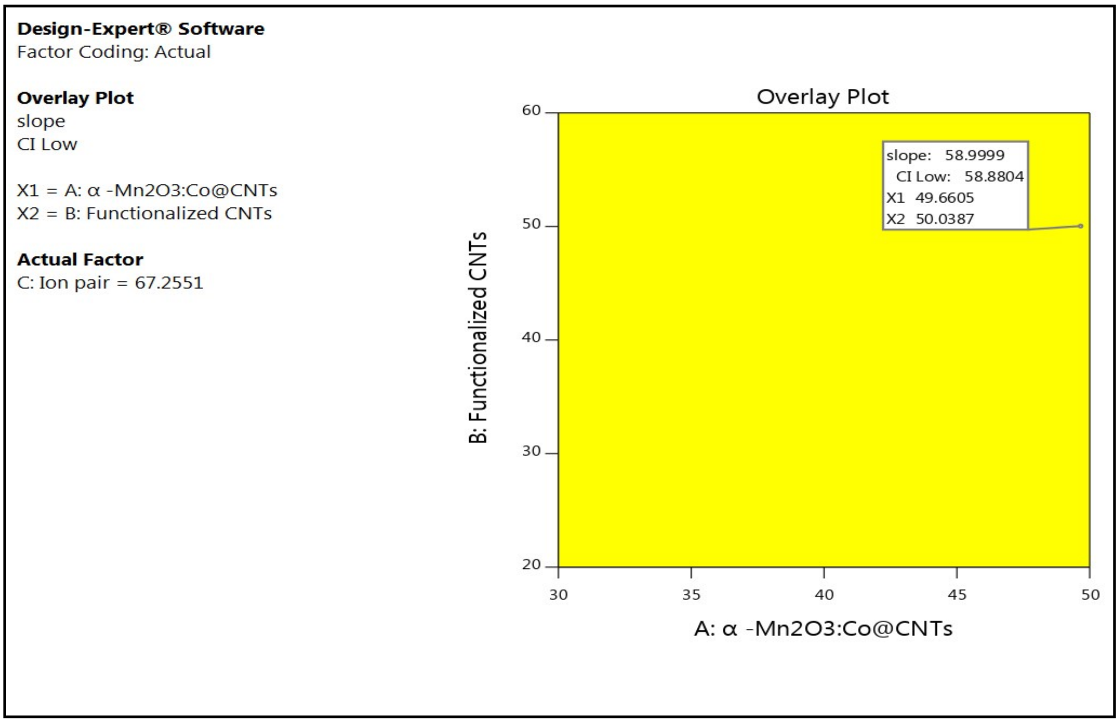

Fig. 3S: overlay desirability plot.

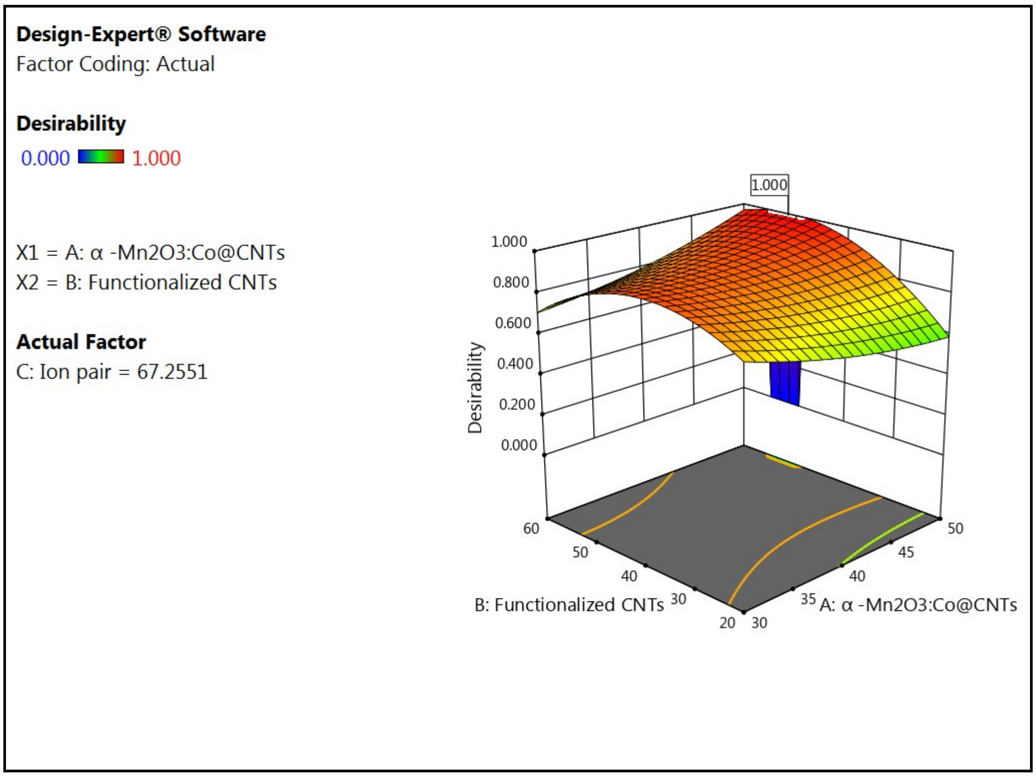

Fig. 4S: 3D desirability plot.
